# Mitochondria-targeted antioxidant MitoQ radiosensitizes tumors by decreasing mitochondrial oxygen consumption

**DOI:** 10.1038/s41420-024-02277-9

**Published:** 2024-12-27

**Authors:** Justin D. Rondeau, Sara Lipari, Barbara Mathieu, Claire Beckers, Justine A. Van de Velde, Lionel Mignion, Mauricio Da Silva Morais, Marvin Kreuzer, Ilaria Colauzzi, Tania Capeloa, Martin Pruschy, Bernard Gallez, Pierre Sonveaux

**Affiliations:** 1 Pole of Pharmacology and Therapeutics, Institut de Recherche Expérimentale et Clinique (IREC), Université catholique de Louvain (UCLouvain), Brussels, Belgium; 2 Biomedical Magnetic Resonance Research Group, Louvain Drug Research Institute (LDRI), UCLouvain, Brussels, Belgium; 3 Laboratory for Applied Radiobiology, Department of Radiation Oncology, University Hospital Zurich, University of Zurich, Zurich, Switzerland; 4 Nuclear and Electron Spin Technologies (NEST) Platform, LDRI, UCLouvain, Brussels, Belgium; 5 WEL Research Institute, WELBIO Department, Wavre, Belgium

**Keywords:** Radiotherapy, Cancer metabolism, Cancer microenvironment, Breast cancer

## Abstract

Hypoxic tumors are radioresistant stemming from the fact that oxygen promotes reactive oxygen species (ROS) propagation after water radiolysis and stabilizes irradiation-induced DNA damage. Therefore, an attractive strategy to radiosensitize solid tumors is to increase tumor oxygenation at the time of irradiation, ideally above a partial pressure of 10 mm-Hg at which full radiosensitization can be reached. Historically, the many attempts to increase vascular O_2_ delivery have had limited efficacy, but mathematical models predicted that inhibiting cancer cell respiration would be more effective. Here, we report that mitochondria-targeted antioxidant MitoQ can radiosensitize human breast tumors in mice. This was not a class effect, as neither MitoTEMPO nor SKQ1 shared this property. At clinically relevant nanomolar concentrations, MitoQ completely abrogated the oxygen consumption of several human cancer cell lines of different origins, which was associated with a glycolytic switch. Using orthotopic breast cancer models in mice, we observed that pretreating hypoxic MDA-MB-231 tumors with MitoQ delayed tumor growth with both single dose irradiation and clinically relevant fractionated radiotherapy. Oxygenated MCF7 tumors were not radiosensitized, suggesting an oxygen enhancement effect of MitoQ. Because MitoQ already successfully passed Phase I clinical trials, our findings foster its clinical evaluation in combination with radiotherapy.

## Introduction

Solid tumors are metabolically heterogeneous, comprising oxidative and glycolytic cancer and host cells. Oxidative cancer cells are typically located close to perfused blood vessels carrying oxygenated red blood cells (RBCs). They are metabolically flexible, using a variety of substrates to fuel the tricarboxylic acid (TCA) cycle and oxidative phosphorylation (OXPHOS) within mitochondria [1]. Comparatively, glycolytic cancer cells primarily populate hypoxic tumor areas, and further comprise proliferating cancer cells that are glycolytic independently of the local partial pressure of oxygen (pO_2_) (the Warburg phenotype) [2, 3]. These cells have less metabolic flexibility because they depend on glucose as an obligatory substrate, but maintain metabolic plasticity as glycolytic and oxidative phenotypes are timely and spatially interchangeable with respect to variations in tumor microenvironment composition [1].

Tumor hypoxia has long been recognized as a major factor limiting cancer cell sensitivity to photon radiotherapy (X- and γ-rays) [4]. It is generally understood that full radiosensitivity can be achieved at a pO_2_ > 10 mm-Hg, with cancer cells at lower pO_2_ levels being increasingly radioresistant [5]. Molecularly, the oxygen enhancement effect (OEE) reflects the involvement of O_2_ in the production and propagation of reactive oxygen species (ROS) following water radiolysis and the ability of O_2_ to stabilize radicals on DNA to produce DNA peroxides [4]. The radiobiological community has therefore deployed intense efforts to find appropriate ways to increase tumor oxygenation at the time of therapeutic irradiation, although with limited success [6, 7]. Among all attempts, only non-invasive hyperthermia, which improves tumor perfusion and inhibits DNA repair, reached clinical use [8, 9].

As an alternative (or in addition) to increasing tumoral oxygen delivery, inhibiting OXPHOS in tumors could also result in enhanced tumor oxygenation at the time of irradiation. According to mathematical models developed by the group of Mark W. Dewhirst [10–12], this strategy is in theory the most effective way to radiosensitize tumors through the OEE, as only a modest decrease in cell respiration could be sufficient to fully alleviate hypoxia. Several drugs have been tested preclinically for this aim, including targeting pathways upstream of OXPHOS (*e.g*., glycolysis with 2-deoxyglucose and the oxidative pathway of lactate with monocarboxylate transporter inhibitors) or the electron transport chain (ETC) either directly or indirectly (*e.g*., Rotenone, Antimycin A, Oligomycin, Metformin, Arsenic trioxide and Insulin) [7, 13, 14]. Intriguingly, examples exist of drugs inhibiting cancer cell respiration in vitro but devoid of any radiosensitizing effects in vivo, with Mito-metformin10 and statins as typical examples [13, 15]. This type of observations emphasizes that in vivo experimentation is mandatory to confirm the radiosensitization properties of each candidate drug.

Over the past 10 years, we have studied mitochondria-targeted antioxidants for their capability to prevent mouse melanoma, human breast cancer and human pancreatic cancer metastasis [16–19]. This family of drugs comprise MitoTEMPO, MitoQ and SKQ1 [20–22], among which MitoQ singles out as it has already successfully passed Phase I safety clinical trials [20]. Molecularly, MitoTEMPO, MitoQ and SKQ1 all comprise a positively charged hydrophobic triphenylphosphonium (TPP^+^) group, a linker, and an antioxidant moiety (ubiquinone in the case of MitoQ) [23]. Previous research demonstrated that the TPP^+^ group allows transmembrane passage and ensures mitochondrial accumulation driven by the mitochondrial potential [24, 25]. Hence, MitoQ is primarily found adsorbed to the inner mitochondrial membrane, where its reduced form (ubiquinol) acts as an antioxidant, becoming oxidized to ubiquinone, which is then reduced by ETC Complex II back to ubiquinol, thus restoring its antioxidant potential [23, 26, 27]. In the cancer cell models that we previously tested, MitoQ inhibited electron leak from the ETC, mitochondrial ROS (mtROS) production, and, consequently, mtROS signaling [16–19]. In this context, we recently showed that MitoQ can inhibit human breast cancer cells migration induced by subclinical doses of photon irradiation [28]. However, whether MitoQ and/or other mitochondria-targeted antioxidants alter tumor radiosensitivity is unknown.

Here, we report that MitoQ dose-dependently inhibits cancer cell respiration, with full inhibition reached at nanomolar doses in several different human cancer cell lines. MitoTEMPO and SKQ1 were devoid of such effects. We further demonstrate that MitoQ can radiosensitize human breast tumors in mice at least partially through the OEE. It did so at a dose of 18 mg/kg *per os* previously reported to be clinically relevant [29].

## Results

### MitoQ reduces the oxygen consumption rate of human cancer cells

**Fig. 1:**
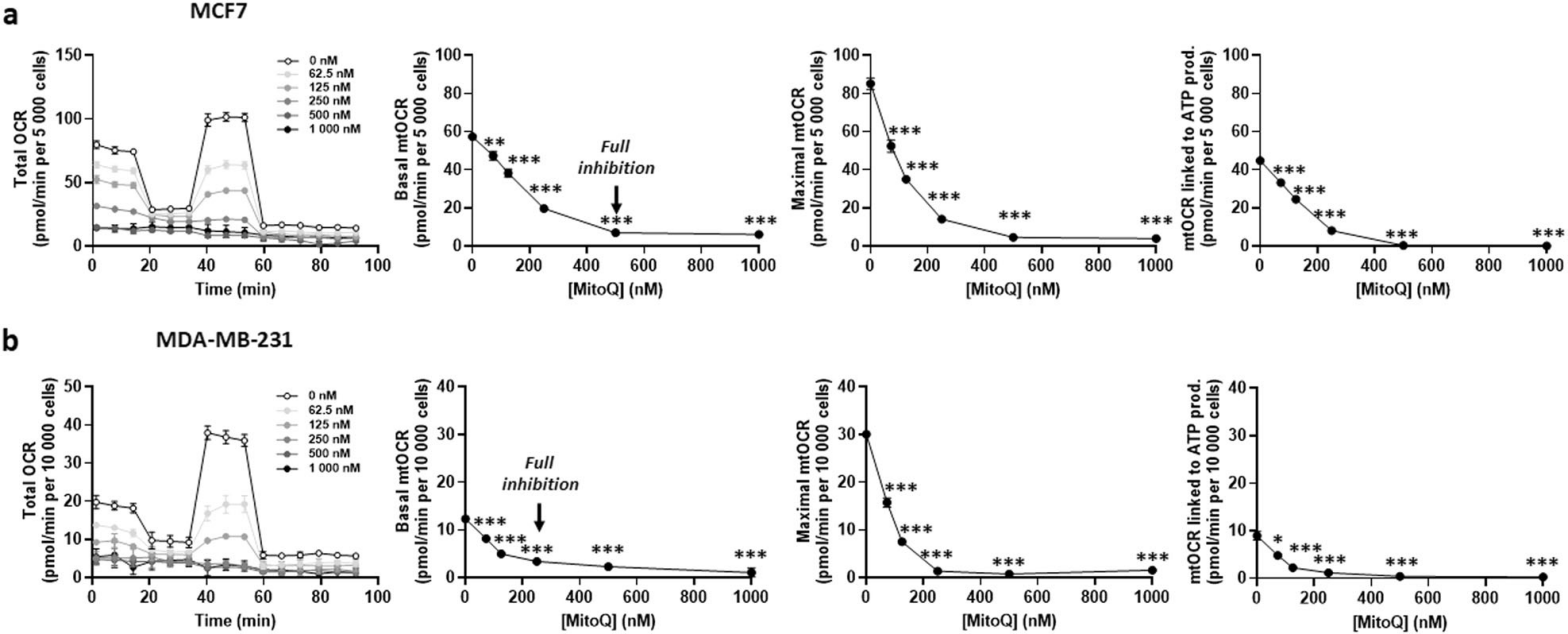
MitoQ dose-dependently reduces human breast cancer cell oxygen consumption rate. **a,** **b** Human breast cancer cells were treated with increasing doses of MitoQ for 24 h. **a** The oxygen consumption rate (OCR) of 5000 MCF7 cells was measured using Seahorse oximetry. The left graph represents total OCR measurements over time with the sequential addition of Oligomycin, FCCP, and Rotenone (Rot) together with Antimycin A (AA). From Seahorse traces, basal, maximal and ATP-linked mitochondrial OCRs (mtOCRs) were calculated (*n* = 4). Full mtOCR inhibition was reached at 500 nM MitoQ (arrow). **b** Same as in **a**, but assessing 10 000 MDA-MB-231 cells. Full mtOCR inhibition was reached at 250 nM MitoQ (arrow). All data are shown as means ± SEM. *P < 0.05, **P < 0.01, ***P < 0.005 by one-way ANOVA with Dunnett’s multiple comparisons test.

To test whether MitoQ could radiosensitize cancer cells, we selected two human breast cancer (BC) cell lines representing different metabolic phenotypes as main models, with MCF7 known to be more oxidative than MDA-MB-231 cells in vitro [30]. MitoQ was tested at doses ranging from 62.5 nM to 1 µM, *i.e*., doses that can be achieved in human plasma and tissues [29], with a readout time of 24 h post-treatment. In 2D culture, MitoQ dose-dependently inhibited the mitochondrial oxygen consumption rate (mtOCR) of both cell lines, similarly affecting basal mtOCR, maximal mtOCR and mtOCR linked to ATP production (Fig. 1). While the smallest tested dose of MitoQ (62.5 nM) already significantly inhibited mitochondrial respiration, full inhibition down to a mtOCR statistically not different from 0 was achieved at doses of 500 nM and 250 nM for MCF7 (Fig. 1a) and MDA-MB-231 (Fig. 1b) cells, respectively.

Based on this observation, we wondered whether other mitochondria-targeted antioxidants could inhibit BC cell respiration. We tested lead compounds MitoTEMPO, whose antioxidant moiety is superoxide dismutase mimetic piperidine nitroxide (TEMPO), and SKQ1, possessing a plastoquinone antioxidant group [22]. Surprisingly, neither MitoTEMPO (Figure S1a) nor SKQ1 (Figure S1b) significantly impacted MCF7 and MDA-MB-231 basal mtOCR, maximal mtOCR or mtOCR linked to ATP production. Thus, among tested mitochondria-targeted antioxidants, only MitoQ was able to induce metabolic oxygen sparing. We therefore reasoned that MitoQ could potentially produce an OEE at clinically relevant doses in the context of radiotherapy.

**Fig. 2:**
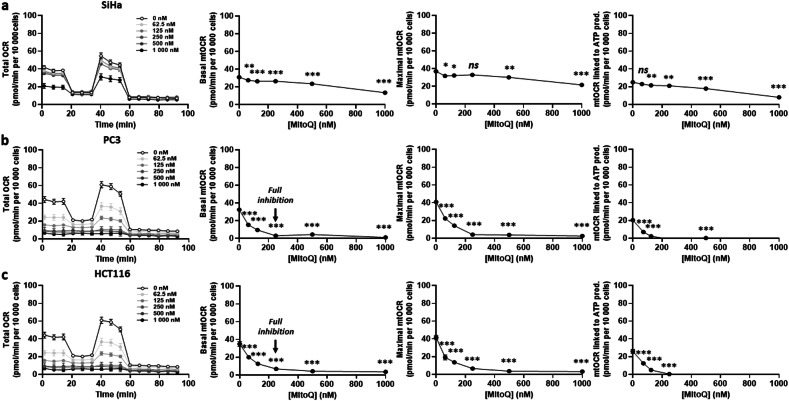
MitoQ dose-dependently reduces the oxygen consumption rate of other human cancer cells. **a**–**c** Cancer cells were treated with increasing doses of MitoQ for 24 h, and the oxygen consumption rate (OCR) of 10 000 cells/well was measured using Seahorse oximetry. Left graphs represent total OCR measurements over time. From Seahorse traces, basal, maximal and ATP-linked mitochondrial OCRs (mtOCRs) were calculated and are displayed on the right. **a** SiHa human cervix cancer cells were tested (*n* = 4). **b** PC3 human prostate cancer cells were tested (*n* = 4). Full mtOCR inhibition was reached at 250 nM MitoQ (arrow). **c** HCT116 human prostate cancer cells were tested (*n* = 4). Full mtOCR inhibition was reached at 250 nM MitoQ (arrow). All data are shown as means ± SEM. *P < 0.05, **P < 0.01, ***P < 0.005 by one-way ANOVA with Dunnett’s multiple comparisons test.

To broaden the scope, we tested additional human cancer cell lines of other origins. MitoQ was capable of inhibiting basal mtOCR, maximal mtOCR and mtOCR linked to ATP production in SiHa human cervix cancer (Fig. 2a), PC3 human prostate cancer (Fig. 2b) and HCT116 human colon cancer (Fig. 2c) cells. Full mtOCR inhibition was reached at a MitoQ concentration of 250 nM in both PC3 and HCT116 cell lines. SiHa cells, which are known to be highly oxidative [31, 32], were comparatively less sensitive, with a ~ 60% inhibition of basal mtOCR reached at 1 µM (Fig. 2a). Overall, all tested human cancer cell lines responded to MitoQ with a decreased mtOCR.

### MitoQ induces a glycolytic switch in human breast cancer cells

**Fig. 3:**
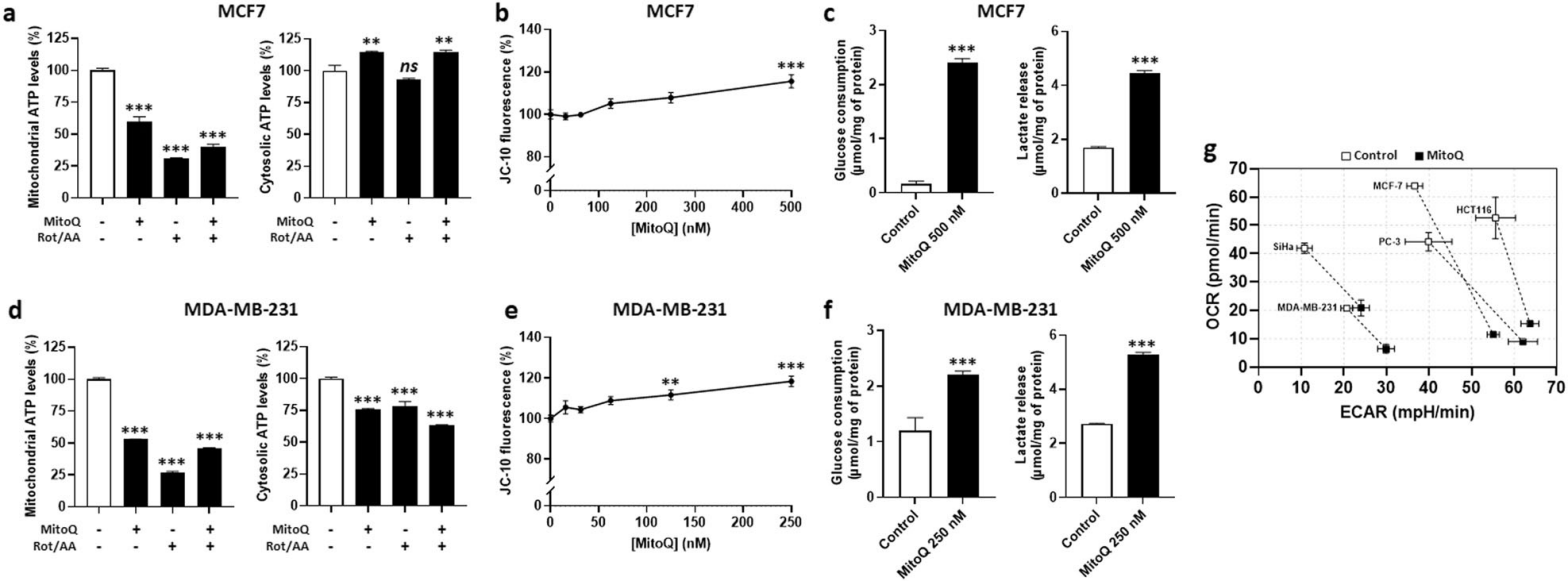
MitoQ induces a glycolytic switch in human breast cancer cells. **a** MCF7 breast cancer cells were treated ± 500 nM MitoQ for 24 h and subjected to subcellular fractionation. Mitochondrial and cytosolic ATP were measured using a fluorescence assay and are reported in the left and right graphs, respectively (*n* = 3). **b** MCF7 cells were treated with increasing doses of MitoQ for 24 h, and ΔΨ was measured *via* JC-10 fluorescence (*n* = 6). **c** Glucose consumption and lactate production rates were measured in MCF7 cells pretreated for 24 h ± 500 nM MitoQ (*n* = 6). **d** As in (a) but using MDA-MB-231 cancer cells treated ± 250 nM MitoQ (*n* = 3). **e** As in (b) but using MDA-MB-231 cells (*n* = 6). **f** As in **c** but using MDA-MB-231 cancer cells treated ± 250 nM MitoQ (*n* = 6). **g** Seahorse XF cell energy map of cancer cells plotted by their basal OCR and basal extracellular acidification rate (ECAR) before and 24 h after treatment with MitoQ at the concentrations identified to bring ATP production linked to mtOCR to 0 (SiHa, 1 µM; MCF7, 500 nM; MDA-MB-231, 250 nM; PC3, 250 nM; HCT116, 250 nM) (*n* = 4–6). All data are shown as means ± SEM. *ns* *P* > 0.05, **P < 0.01, ***P < 0.005 compared to untreated controls; by one-way ANOVA with Tukey’s multiple comparisons test (**a, b, d, e**) or Student’s t test (**c, f**).

In MCF7 cells, OXPHOS inhibition by 500 nM MitoQ decreased mitochondrial ATP production to the same extent as a treatment with 0.5 µM Rotenone + 0.5 µM Antimycin A, but the effects were not additive (Fig. 3a). Mitochondrial potential (ΔΨ) was nevertheless dose-dependently increased (Fig. 3b), reflecting the accumulation of positively charged MitoQ at the inner mitochondrial membrane [33]. Upon OXPHOS inhibition, the initially highly oxidative MCF7 cells switched to a more glycolytic metabolism characterized by enhanced glucose uptake and lactate release (Fig. 3c) with increased cytosolic ATP production (Fig. 3a). Like MCF7, MDA-MB-231 cells treated with 250 nM MitoQ had reduced mitochondrial ATP production (Fig. 3d) despite increased ΔΨ (Fig. 3e). These cells also had increased glucose uptake and lactate release (Fig. 3f), but this effect was not sufficient to prevent a drop in cytosolic ATP levels (Fig. 3a).

To better estimate the amplitude of the glycolytic switch, we measured extracellular acidification rates (ECAR) at the precise MitoQ doses that optimally inhibited mtOCR. MitoQ decreased the OCR (Figs. 1 and 2) and increased the ECAR (Fig. S2) of all tested cell lines, confirming a MitoQ-induced glycolytic switch. Figure 3g depicts the glycolytic switch in MCF7, MDA-MB-231, SiHa, PC3 and HCT116 human cancer cells.

Conclusively at this stage, our data showed that MitoQ dose-dependently reduces cancer cell respiration, which involves a compensatory increase in their glycolytic rate.

### MitoQ radiosensitizes hypoxic human breast cancer cell in vitro

**Fig. 4:**
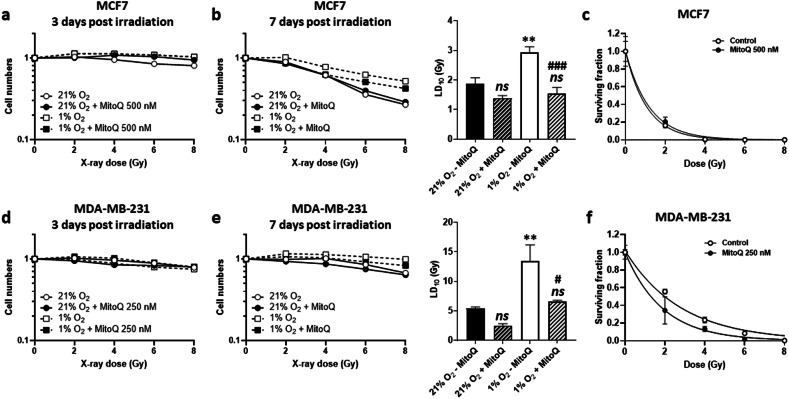
MitoQ radiosensitizes hypoxic human breast cancer cell in vitro. **a** MCF7 human breast cancer cells were treated ± 500 nM MitoQ for 24 h under normoxia (21% O_2_) or hypoxia (1% O_2_). On the next day, cells were irradiated with increasing doses. Cells were then maintained under normoxia or hypoxia until surviving fractions were determined (**a**) 3 days (*n* = 5) or (**b**) 7 days post irradiation. LD_10_ for day 7 are displayed in the graph on the right (*n* = 5). **c** MCF7 cells were pretreated ± 500 nM MitoQ for 24 h, irradiated with increasing doses, and tested for clonogenicity. The graph shows the surviving fraction as a function of the irradiation dose (*n* = 3). **d** As in **a** but using MDA-MB-231 human breast cancer cells treated ± 250 nM MitoQ (*n* = 6). **e** As in **b** but using MDA-MB-231 cells treated ± 250 nM MitoQ (*n* = 5-6). **f** As in **c** but using MDA-MB-231 cells treated ± 250 nM MitoQ (*n* = 3). All data are shown as means ± SEM. *ns* P > 0.05, **P < 0.01 compared to the normoxic control; ^#^P < 0.05, ^###^P < 0^.^001 compared to hypoxia alone; by one-way ANOVA with Tukey’s multiple comparisons test (**b, d**) or Student’s t test (**e**).

A potential OEE in response to MitoQ was tested by comparing cancer cells treated ± MitoQ under normoxia (21% O_2_) and hypoxia (1% O_2_). Under normoxia, a pretreatment with MitoQ (500 nM for 24 h) did not impact MCF7 cell number at 3 days (Fig. 4a) or 7 days post-irradiation (Fig. 4b). Under hypoxia, MitoQ radiosensitized hypoxic MCF7 cells, which was seen 7 days post-irradiation; while hypoxia significantly increased the irradiation dose necessary to kill 10% of cells (LD_10_), MitoQ reduced the LD_10_ of hypoxic MCF7 cells down to levels seen in normoxia (Fig. 4b). However, this was not sufficient to reduce clonogenic survival after hypoxia (Fig. 4c).

Similarly to MCF7 cells, MitoQ (250 nM for 24 h) did not alter the radiosensitivity of normoxic MDA-MB-231 cells (Fig. 4d). However, it radiosensitized hypoxic MDA-MB-231 cells, which was seen both by analyzing cell number 7 days post-irradiation (Fig. 4e) and by clonogenic survival (Fig. 4f). We therefore concluded that MitoQ can radiosensitize MDA-MB-231, but not MCF7 cancer cells, possibly through the OEE, which we next tested in 3D.

### MitoQ decreases hypoxia in human breast cancer spheroids

**Fig. 5:**
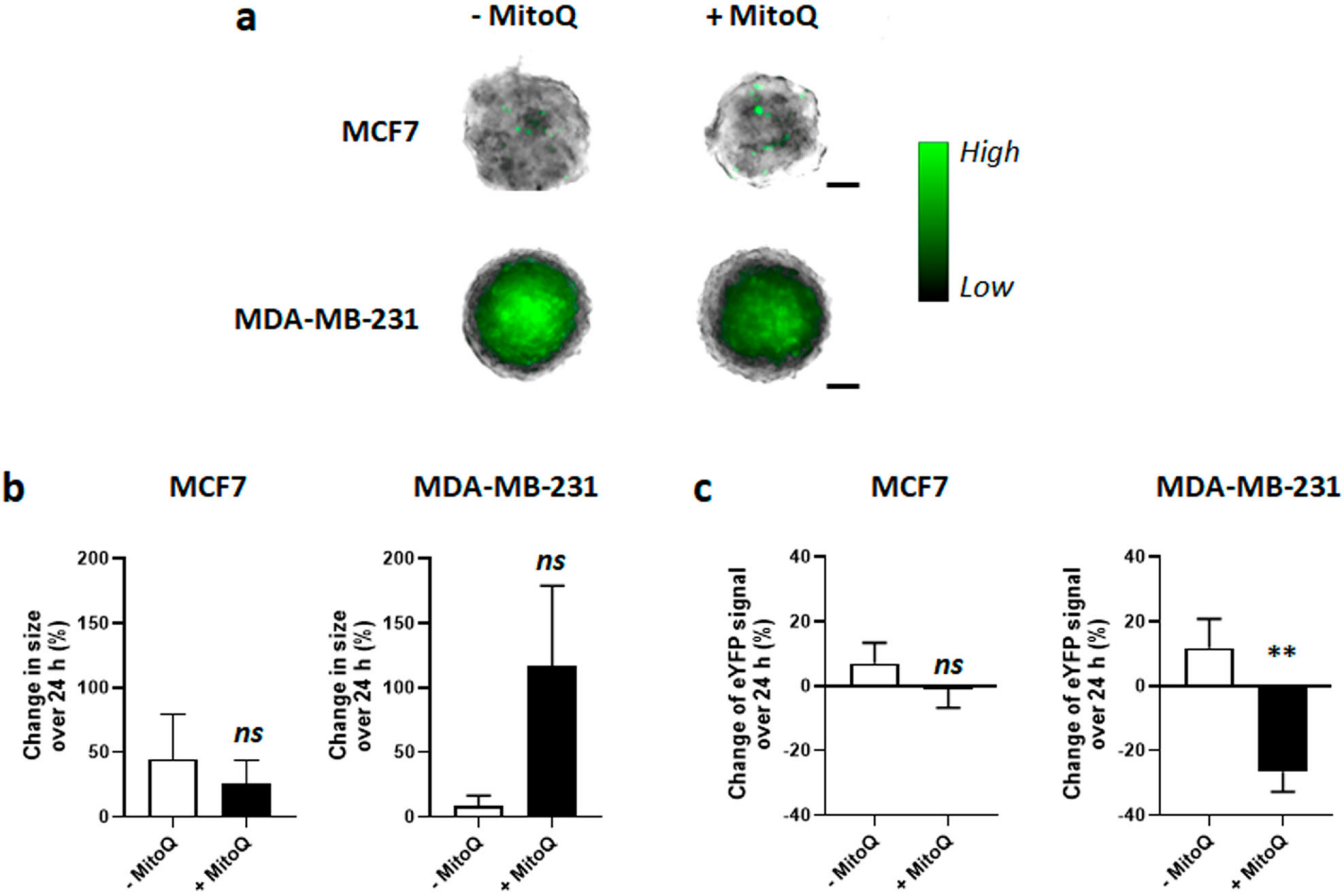
MitoQ increases MDA-MB-231 spheroid oxygenation. **a–c** HIF-TK-eYFP spheroids were treated ± MitoQ for 24 h (500 nM for MCF7 and 250 nM for MDA-MB-231 cells). **a** Representative microscopy images of MCF7 and MDA-MB-231 spheroids after treatment, where the eYFP signal appears in green (scale bar = 100 µm). A color bar is provided. **b** MCF7 (left graph, *n* = 7-8) and MDA-MB-231 (right graph, *n* = 8) spheroid size. **c** Quantification of MCF7 (left graph, *n* = 7-8) and MDA-MB-231 (right graph, *n* = 8) eYFP expression reporting on HIF activity. All data are shown as means ± SEM. *ns* P > 0.05, **P < 0.01, by Student’s t test **(b, c)**.

Spheroids are known to have an inner hypoxic core resulting from the respiration of outer cell layers. To test reoxygenation by MitoQ, we used a hypoxia-inducible factor (HIF)-binding sites-thymidine kinase (TK) promoter-fluorescent enhanced yellow fluorescent protein (eYFP) construct (HIF-TK-eYFP) reporting on the activity of hypoxia-inducible factors HIF-1 and HIF-2 [34]. MitoQ at the aforementioned concentrations produced no change in spheroid size after 24 h of treatment (Fig. 5a, b), but significantly decreased MDA-MB-231 HIF-TK-eYFP fluorescence by ~30%, while in MCF7 there was no change (Fig. 5a, c). Of note, basal differences in eYFP signal intensity between the two types of spheroids can be attributed to the fact that MDA-MB-231 cells form tight spheroids with a true hypoxic core, whereas MCF7 cells form homogenously loose clusters of cells as spheroids, more permeable to oxygen [35]. Although our results suggested that MitoQ can reoxygenate glycolytic tumor spheroids, at this stage they remained inconclusive with regard to hypoxia because HIF activity not only depends on microenvironmental pO_2_ but also on confounding factors, including redox state [36, 37] and intracellular pyruvate levels [38].

### MitoQ radiosensitizes MDA-MB-231 human breast tumors in mice

**Fig. 6:**
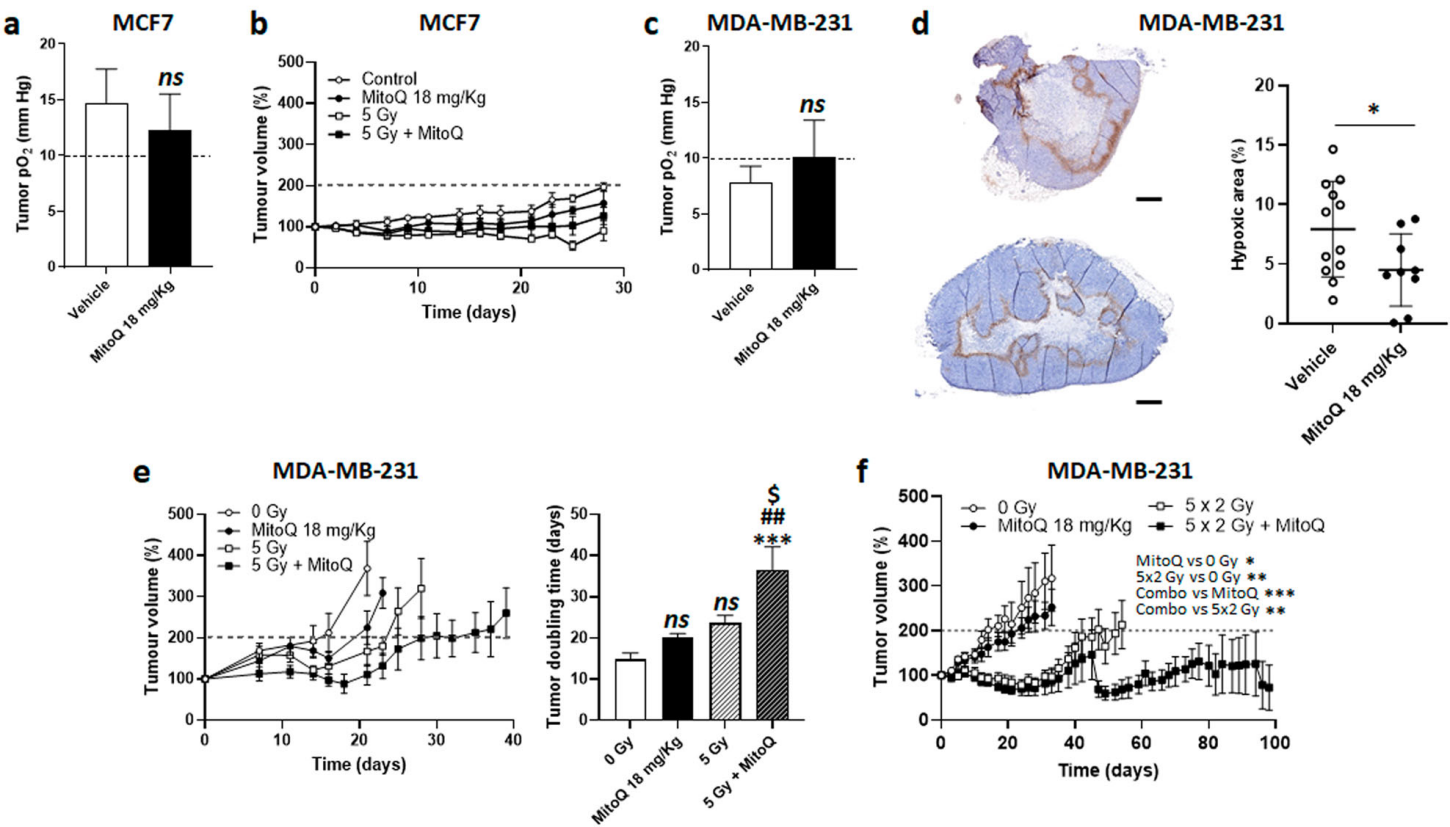
MitoQ radiosensitizes human breast tumors in mice. **a–f** Human breast cancer cells were implanted orthotopically in the breast of immunodeficient mice. When average tumor volumes reached 100 mm^3^ (Day -1), mice were randomly assigned to a treatment group. **a** On Day -1, MCF7-bearing mice received MitoQ (18 mg/kg *per os*) or saline. MCF7 tumor pO_2_ was measured using electron paramagnetic resonance (EPR) after treatment on Day 0 (*n* = 7–8). **b** On Day -1, MCF7-bearing mice received MitoQ (18 mg/kg *per os*) or saline. On Day 0, a local 5 Gy or a sham irradiation was delivered to mice. The graph shows tumor growth over time with the dashed line representing tumor volume doubling (*n* = 3). **c** As in **a** but using MDA-MB-231 tumors in mice (*n* = 8–9). **d** On Day -1, MDA-MB-231-bearing mice received MitoQ (18 mg/kg *per os*) or not. On Day 0, all mice received an intraperitoneal injection of pimonidazole (60 mg/kg) and were sacrificed 90 min later. Representative pictures are shown on the left, where pimonidazole staining indicating hypoxia appears in brown. Pimonidazole-positive area normalized for total tumor area is shown in the right graph, where each dot represents a mouse tumor (*n* = 9–12, scale bars = 500 µm). **e** As in **b**, but using MDA-MB-231 tumors in mice. The graph on the left shows tumor growth over time. Tumor doubling times are shown on the right graph (*n* = 7-8 per group). **f** MDA-MB-231 tumor-bearing mice received MitoQ (18 mg/kg *per os*) for 5 consecutive days (from Day -1 to Day +3; 5 doses in total) or not. Radiotherapy or sham irradiation was delivered 24 h after each dose of MitoQ, i.e., from Day 0 to Day +4 (5 × 2 Gy). The graph shows tumor growth over time (*n* = 8–9 per group) with the dashed line representing tumor doubling. All data are shown as means ± SEM. *ns* *P* > 0.05, *P < 0.05 by Student’s t test (**a, c, d**). *ns* P > 0.05, ***P < 0.005 compared to 0 Gy; ^##^P > 0.01 compared to MitoQ without irradiation; ^$^P < 0.05 compared to irradiation without MitoQ; by one^-^way ANOVA with Tukey’s multiple comparisons test (**e**) ; * P < 0.05, **P < 0.01, ***P < 0.001 by two-way ANOVA (**f**).

We finally aimed to test whether MitoQ could radiosensitize orthotopic human breast tumors in mice. For MCF7, electron paramagnetic resonance (EPR) oximetry revealed a basal tumor oxygenation above 10 mm-Hg, which a single clinically relevant dose of MitoQ (18 mg/kg *per os*) did not increase (Fig. 6a). In line with this observation, MitoQ did not radiosensitize MCF7 tumors in mice (Fig. 6b). In MDA-MB-231 tumors in mice, EPR was not sensitive enough to detect a significant increase in pO_2_ after a single dose of MitoQ (Fig. 6c). To better detect hypoxia, we therefore used immunohistochemistry with pimonidazole, which revealed a significant decrease in MDA-MB-231 tumor hypoxia 24 h after MitoQ administration (Fig. 6d). Of note, a statistical outlier was excluded from the MitoQ group. Tumor reoxygenation was associated with effective tumor radiosensitization using a single dose of radiotherapy (5 Gy), with measured tumor doubling times of 14.9 ± 1.5 days in vehicle-treated mice, 20.1 ± 1.0 days for MitoQ alone (+ 5.2 days, P > 0.05), 23.7 ± 1.8 days with a single dose irradiation of 5 Gy (+ 8.8 days, P > 0.05), and 36.3 ± 5.8 days for the combination treatment (+ 21.4 days, P < 0.005) (Fig. 6e). Of note, a statistical outlier was excluded from the 5 Gy group and another from the combination group.

This prompted us to test a fractionated radiotherapy regimen. MDA-MB-231 tumor-bearing mice were locally irradiated with a daily dose of 2 Gy for 5 consecutive days, with or without MitoQ administration as a pretreatment prior to each irradiation. Measurements of tumor growth revealed that the combination treatment was more effective than individual treatments (Fig. 6f). One hundred days after treatment, tumor doubling times were not reached in mice treated with 5 x MitoQ + 5 × 2 Gy, while mean doubling times were 11.5 ± 0.6 days in the control group, 19.4 ± 3.3 days in the 5 x MitoQ group (P > 0.05 compared to control), and 52.4 ± 6.8 days in the 5 × 2 Gy group (P < 0.01 compared to control). Of note, one mouse was excluded in the 5 × 2 Gy group due to an ascites unrelated to the experiment. Whole curve analyses confirmed that the combination treatment was significantly more effective that MitoQ alone (*P* < 0.001) or fractionated radiotherapy alone (P < 0.01). Interestingly, chronic MitoQ administration for 5 days modestly but significantly (*P* < 0.05) delayed tumor growth in the absence of irradiation. Furthermore, vimentin staining of the tumors that never reached the doubling endpoint in the combination group revealed the presence of remaining cancer cells (Figure S3), indicating that longer treatments times would be required to potentially eradicate orthotopic MDA-MB-231 tumors in mice.

## Discussion

We report that MitoQ radiosensitizes tumors at least partially through the OEE, as it radiosensitized hypoxic but not normoxic cancer cells and reduced tumor hypoxia in vivo.

In this study, we observed that MitoQ dose-dependently inhibited the respiration of different types of human cancer cells. This was unexpected because the reported mode of action of MitoQ is classically that of an antioxidant selectively inactivating mtROS [39]. Our data suggest another mode of action through which MitoQ, inserted into the inner mitochondrial membrane [29, 40], blocks electron flux through the ETC, resulting in O_2_ sparing. Similarly to Coenzyme Q10, MitoQ can accept electrons from Complexes I and II [29]. However, due to steric hindrance of the TPP^+^ group [27], MitoQ would not be able to transfer these electrons to Complex III and thus block electron flux through the ETC [27]. Of note, we verified that MitoQ did not replace O_2_ as a final electron acceptor at Complex IV, which was evidenced by showing that increasing doses of MitoQ did not generate increasing levels of mitochondrial ATP. MitoQ at nanomolar doses (250 - 500 nM) rather decreased mitochondrial ATP generation in human BC cells to the same extent as Rotenone + Antimycin A. Cells compensated by increasing glycolytic ATP production, which could explain why MitoQ exerts cytostatic but not cytotoxic effects on human BC cells [18] as well as the modest growth delay observed upon chronic mouse treatment with MitoQ alone.

Among tested mitochondria-targeted antioxidants, only MitoQ dose-dependently decreased cancer cell respiration, whereas MitoTEMPO and SKQ1 had modest effects that were not dose-dependent. This observation rules out a class effect, which could be explained by different localizations of the mitochondria-targeted antioxidants with respect to the inner mitochondrial membrane and ETC complexes as well as different antioxidant capacities and turnover rates on target. To our knowledge, redox recycling involving Complex II has been reported for MitoQ [27], but not for other mitochondria-targeted antioxidants. This would support the dose-dependent inhibition of cell respiration that we observed, as a single molecule of MitoQ could sequentially and repeatedly capture electrons that would be transferred to electron acceptors other than O_2_. The paradoxical effect of MitoQ on ΔΨ (a decrease was expected but an increase was observed) has been reported by others: MitoQ creates a pseudo-mitochondrial membrane potential as the positively charged molecule (TPP^+^ group) accumulates at the inner mitochondrial membrane [40].

According to mathematical models [10–12], the most adequate mode for tumor reoxygenation is by inhibiting cancer cell respiration rather than increasing vascular O_2_ delivery. However, not all drugs that inhibit cancer cell respiration in vitro can radiosensitize tumors [13, 15], which could be linked to issues involving drug delivery, biodistribution, stability in biological systems, metabolism, clearance, and/or on- and off-target effects impacting the radiotherapeutic response. Thus, associating a reduced mtOCR in vitro to in vivo tumor radiosensitization is not straightforward. Here, we observed that MitoQ can radiosensitize tumors in vivo when using single-dose radiotherapy and more clinically relevant fractionated mode radiotherapy. Radiosensitization was seen when the tumors were hypoxic (MDA-MB-231 model) but not when they had basal oxygen levels above 10 mm-Hg (MCF7 model), further supporting that MitoQ acts, at least in part, through the OEE. The inability of glycolytic MDA-MB-231 cancer cells to rescue global ATP production through further increasing the glycolytic rate, while MCF7 cells succeeded, may further contribute to radiosensitization, as energy is necessary for repair. We did not test MitoTEMPO nor SKQ1 in vivo, as these drugs minimally influenced cancer cell respiration in vitro.

In vitro, maximal OCR inhibition 24 h after a single dose of MitoQ was reached at doses ranging from 250 nM to 1 µM. This range is close to the MitoQ concentrations measured in vivo after bolus delivery. In mice, we previously reported that free MitoQ reached a plasma concentration of ~50 nM 4 h after the administration of 12 mg/kg to 24 mg/kg *per os* [18]. A similar ~50 nM concentration was achieved in human plasma 1 h after a dose of 1 mg/kg MitoQ *per os* [29]. In mouse tissues, MitoQ concentrations reached ~100 nmol/kg in the lungs, between ~50 and ~100 nmol/kg in the heart, and between ~400 nmol/kg and ~1.2 µmol/kg in the liver and kidneys. Since MitoQ accumulates in mitochondria and because several doses should be delivered in the context of fractionated radiotherapy, we believe that our observations are clinically relevant.

Although there is previous in vitro evidence that MitoQ could display cancer cell selectivity (which has been purported to be based on higher mitochondrial membrane potential in cancer compared to nonmalignant cells) [18], selectivity is not needed in the context of radiotherapy because normal tissues already have a pO_2_ above 10-mm Hg and therefore cannot be further radiosensitized through the OEE. Rather, several groups previously showed that MitoQ acts as a radioprotective agent for normal tissues, including cultured human astrocytes [41], mouse intestines [42], and rat testis [43]. In rat testis, MitoQ promoted mitochondrial ATP production [43], whereas it reduced ATP production by cancer cells (our study). In mouse intestines, MitoQ inhibited mitochondria-dependent apoptosis and inflammation [42]. Whether these differences between normal and tumor tissues are driven by the lower mitochondrial potential of nonmalignant cells compared to metabolically hyperactive cancer cells warrants further investigation.

Although we report that MitoQ can radiosensitize breast tumors *via* the OEE, additional mechanisms are possible. Autophagy, which is well-known to modulate radiosensitivity [44], is one among them. Previous studies in nonmalignant cells have indeed reported that MitoQ can stimulate autophagy, either through caspase-1 activation following thioredoxin-interacting protein binding to NOD-like receptor family pyrin domain containing 3 [45] or by an AMP-activated protein kinase-regulated mechanism [41]. Likewise, in the context of osteoarthritis it was found that MitoQ activates a nuclear factor erythroid 2-related factor 2-Parkin-regulated mitophagy program in chondrocytes [46]. Interestingly, MitoQ-induced autophagy was proposed to originate from the creation of a pseudo-mitochondrial membrane potential when the positively charged drug accumulates within mitochondria [40]. To our knowledge, whether MitoQ also triggers autophagy to radiosensitize tumors is unknown.

Conclusively, our study shows that MitoQ is a tumor radiosensitizer, acting at least partially though the OEE. This is not a class effect, as the other mitochondria-targeted antioxidants that we tested did not inhibit OXPHOS in cancer cells.

## Materials and Methods

### Ethical statement

All mouse experiments were performed with the approval of UCLouvain *Comité d’Ethique pour l’Expérimentation Animale* (approval ID: 2020/UCL/MD/033) according to national and European animal care regulations.

### Cells and cell culture

MCF7 (American Type Culture Collection [ATCC], Manassas, VA, USA; catalog #HTB-22) and MDA-MB-231 (ATCC, catalogue #HTB-26) human breast adenocarcinoma cancer cells, SiHa human cervix cancer cells (ATCC, catalog #HTB-35), PC3 human prostate cancer cells (ATCC, catalog #CRL-1435) and HCT116 human colon cancer cells (ATCC, catalog #CCL-247) were cultured at 37 °C in a 5% CO_2_ humidity-controled incubator in DMEM containing GlutaMAX, 4.5 g/L *D*-glucose without pyruvate (ThermoFisher Scientific, Dilbeek, Belgium; catalogue #10566016), supplemented with 10% FBS (Sigma-Aldrich, Overijse, Belgium). Cell authenticities were routinely verified with short tandem repeat testing (Eurofins Genomics, Ebersberg, Germany). Cultured cells were routinely checked for mycoplasma contamination (MycoAlert Plus, Lonza, Verviers, Belgium; catalog #LT07-710).

### Cells, cell treatments and numbering

Where indicated, cells were treated with Mitoquinol mesylate (MitoQ) [47], (2-(2,2,6,6-tetramethylpiperidin-1-oxyl-4-ylamino)-2-oxoethyl)triphenylphosphonium chloride (MitoTEMPO; Sigma-Aldrich catalog #SML0737) or Visomitin (SKQ1; MedChemExpress, Sollentuna, Sweden; catalog #HY-100474). Hypoxia (1% O_2_) was achieved by a 24 h incubation in a Whitley H35 hypoxystation (Don Whitley Scientific, Bingley, UK). Subconfluent cells were irradiated at a dose rate of 0.8 Gy/min using an IBL-637 ^137^Cs γ-ray irradiator (Gamma Service Medical, Leipzig, Germany), and spheroids at a dose rate of 2.132 Gy/min using a RS-2000 225 kV irradiator (Rad Source Technologies, Buford, GA, USA). Cell numbers were determined on a SpectraMax i3x spectrophotometer equipped with a MiniMax imaging cytometer (Molecular Devices, San Jose, CA, USA).

### Metabolic assays

Cellular OCRs and ECARs were determined on a Seahorse XFe96 bioenergetic analyzer, glucose and lactate levels in cell supernatant using enzymatic assays on an ISCUS^flex^ CMA600 bioenergetic analyzer (Aurora Borealis Control, Schoonebeek, The Netherlands) [31], mitochondrial and cytosolic ATP measurements using a CellTiter-Glo 2.0 Cell Viability Assay (Promega, Leiden, The Netherlands; catalog #G9243) after subcellular fractionation, and ΔΨ using a JC-10 Mitochondrial Membrane Potential Assay Kit (Abcam, Cambridge, UK; catalogue #ab112134). Details of the procedures are available in the Supplementary Information.

### Clonogenic assays

Cells were pre-treated for 24 h ± MitoQ, irradiated or not, and seeded in 6-well plates (1 000 cells/well). Two weeks later, they were fixed and stained for 1 h with 0.5% crystal violet in a 10% ethanol solution. Colonies were washed with water and counted. Results are expressed as surviving fraction (SF), where SF = #colonies/plating efficiency (PE).

### Spheroids

Cells were stably infected with an HBR-6U lentiviral vector expressing eYFP under the control of six hypoxia-responsive element (HRE) repeats (Addgene, Watertown, MA, USA; plasmid #42621) [34]. After transduction, cells were cultured in hypoxic conditions (1% O_2_) and selected by fluorescence-activated cell sorting (FACSAriaTM III Cell Sorter, BD Bioscience), as previously reported [48]. After recovery in normoxia for one week, 5000 cells/well were plated, supplemented with 15 µg/ml of rat tail collagen I (ThermoFisher Scientific, catalog #A1048301) 24 h later, and allowed to form spheroids. Spheroids were imaged on the next day before MitoQ administration, treated ± MitoQ, and 24 h after MitoQ administration. All images were captured on a Leica widefield Dmi8 microscope with the same settings, quantified using the FIJI ImageJ software with a custom-written script, and analyzed according to a previously described protocol [48].

### In vivo experiments

All in vivo experiments were performed on 5-week-old female Rj:NMRI-Foxn1 nu/nu mice (Janvier Labs, Le Genest-Saint-Isle, France). Orthotopic tumor implantation was performed under anesthesia (80 mg/kg of ketamine and 8 mg/kg of xylazine) as previously described [17]. Tumor size was then monitored every two days using an electronic caliper. When average tumor volumes reached 100 mm^3^ (Day -1), mice were randomly assigned to a treatment group. On that day, MitoQ at a dose 18 mg/kg [29] or an equal volume of saline was administered by gavage to the indicated animal subgroups. There was not blinding. Four independent series of experiments were conducted to determine tumor pO_2_ using EPR oximetry, to quantify tumor hypoxia using pimonidazole hydrochloride (MedChemExpress catalog #HY-105129), and to evaluate the radiosensitizing potential of MitoQ in animals treated with single dose (5 Gy) or repeated doses (5 × 2 Gy) of radiotherapy at 0.8 Gy/min using an IBL-637 ^137^Cs γ-ray irradiator. Details of the procedures are available in the Supplementary Material. The ethical endpoint for tumor size was 1000 mm^3^, which was not exceeded. For a significant Cohen effect of 2.0 at the alpha threshold of 0.05 and with a power of 90%, we used minimum 6 animals per group. Animals for which tumor implantation failed and statistical outliers were excluded from analysis.

### Statistics

All results are presented as means ± standard error of the mean (SEM) for *n* independent observations. Error bars are sometimes smaller than symbols. Outliers were identified using Dixon’s Q test for all data except for tumor growth delays for which the robust regression followed by outlier identification (ROUT) method was used with a 1% Q. All data were analyzed using GraphPad Prism 10.2.3 considering similar variance between the groups. Two-sided Student’s *t* test, one-way ANOVA with Dunnett’s or Tukey’s post-hoc test and two-way ANOVA were used where appropriate. P < 0.05 was considered to be statistically significant.

## Supplementary information

Supplementary data

## Data Availability

All data generated or analyzed during this study are included in this article and its supplementary information file.
