# Supplementary material for: Mitochondria-targeted antioxidant MitoQ radiosensitizes tumors by decreasing mitochondrial oxygen consumption
Source: Cell Death Discov. 2024 Dec 27;10:514. doi: 10.1038/s41420-024-02277-9 (PMC11681259; doi:10.1038/s41420-024-02277-9)
Supplement: Supplementary file 1 — Supplementary data [file 41420_2024_2277_MOESM1_ESM.docx]

**Mitochondria-targeted antioxidant MitoQ radiosensitizes tumors**

**by decreasing mitochondrial oxygen consumption**

**Supplementary Materials and Methods**

**Seahorse oximetry and pH-metry.** Cellular OCRs and ECARs were determined on a Seahorse XFe96 bioenergetic analyzer using the XF cell MitoStress kit (Agilent Technologies, Zaventem, Belgium), according to the manufacturer’s protocol. Briefly, 10 000 MCF-7, SiHa, PC3 or HCT116 cells or 5 000 MDA-MB-231 cells/well were seeded in XF96 culture plates in their regular culture medium ± MitoQ, MitoTEMPO or SKQ1. The next day, culture medium was replaced by DMEM containing 10 mM glucose, 2 mM glutamine, 1.85 g/L NaCl, and 3 mg/L phenol red, pH 7.4. Cells were incubated for 1 h in a CO_2_-free incubator before analysis. Basal mtOCR was calculated by subtracting OCR after the addition of 0.5 µM Complex I inhibitor Rotenone together with 0.5 µM Complex III inhibitor Antimycin A from OCR without treatment; maximal mtOCR by subtracting OCR after the addition of Rotenone and Antimycin A from OCR after the addition of 1 µM ionophore carbonyl cyanide-4-(trifluoromethoxy)phenylhydrazone (FCCP), and ATP-linked mtOCR by subtracting OCR without any treatment from OCR after the addition of 1 µM ATP synthase inhibitor oligomycin. All data were normalized to cell numbers determined on a SpectraMax i3x spectrophotometer equipped with a MiniMax imaging cytometer (Molecular Devices).

**Glucose consumption and lactate release assays.** Glucose and lactate levels were determined over time in cell supernatant using enzymatic assays on an ISCUS^flex^ CMA600 bioenergetic analyzer (Aurora Borealis), as previously reported [1]. All data were normalized by total protein content determined using the Pierce BCA Protein Assay (ThermoFisher Scientific catalog #23224 and 23228), according to manufacturer’s instructions.

**Mitochondrial and cytosolic ATP measurements.** Cells (2 x 10^7^ per condition) were pre-treated for 24 h ± MitoQ and for 6 h ± 0.5 µM Complex I inhibitor Rotenone together with 0.5 µM Complex III inhibitor Antimycin A before pellet collection. Subcellular fractionation was performed using the Mitochondria Isolation Kit for Cultured Cells (ThermoFisher Scientific, catalog #89874) according to manufacturer’s protocol. ATP levels in mitochondrial and cytosolic fractions were measured using a CellTiter-Glo 2.0 Cell Viability Assay (Promega catalog #G9243) on a SpectraMax i3x spectrophotometer.

**Mitochondrial membrane potential.** ΔΨ was measured using a JC-10 Mitochondrial Membrane Potential Assay Kit (Abcam catalogue #ab112134), following manufacturer’s instructions. Briefly, 10 000 MCF7 or 5 000 MDA-MB-231 cells/well in 96-well plates were treated for 24 h ± MitoQ. After treatment, cells were washed twice with PBS and stained with JC-10 for 45 min. Fluorescence intensities were read using a SpectraMax i3 spectrophotometer.

***In vivo* experiments.** A first series of animals was used to determine tumor pO_2_ using EPR oximetry on Day 0 and on Day +1. A previously disclosed protocol was used on mice anesthetized with 3% isoflurane for 10 min and maintained at 37°C with 1.5% isoflurane on a heating blanket, with lithium phthalocyanine crystals as the oxygen sensor [2]. After EPR oximetry, mice were sacrificed using terminal anesthesia and cervical dislocation. Tumor oxygen concentrations were calculated as a function of the EPR linewidth calibrated to pO_2_ levels.

A second series of animals was used to determine tumor hypoxia on Day +1. On that day, mice were given an intraperitoneal injection of 60 mg/kg pimonidazole hydrochloride (MedChemExpress catalog #HY-105129) in PBS and sacrificed 90 min later using terminal anesthesia and cervical dislocation. Tumors were collected, fixed in 4% PFA and embedded in paraffin. Tumors were cut using an Epredia HM 355S automated microtome (ThermoFisher Scientific), and 5 µm-thick slices were mounted onto glass slides, deparaffinized, and blocked using 5% bovine serum albumin (BSA) in tris-buffered saline (TBS, 45 g NaCl, 6.05 g Tris base in 5 L ddH_2_O, pH 7.2-7.4) with 0.1% Tween 20. Slides were then stained with an anti-pimonidazole antibody (Hypoxyprobe, Burlington, MA, USA; catalog #PAb2627) in 1% BSA for 1 h and incubated with an anti-rabbit HRP secondary antibody (Agilent Technologies catalog #K4003) for 40 min. They were further incubated in a 3,3′-Diaminobenzidine (DAB) staining solution (Agilent Technologies catalog #K3468) for 5 min and counterstained with hematoxylin (Agilent Technologies catalog #S3301) for 5 min. After washing and drying, coverslips were mounted using an automated coverslipper (Sakura Finetek, Berchem, Belgium) and imaged using a Panoramic Scan II slide scanner (3D HisTech). Pimonidazole-positive area and total tumor area were quantified using the QuPath software.

A third series of animals received a single local 5 Gy or sham irradiation on Day 0, at 0.8 Gy/min using an IBL-637 ^137^Cs γ-ray irradiator, *i.e.*, 24 h after MitoQ treatment. Tumor growth was then monitored over time using an electronic caliper to establish growth delay curves as previously shown [3]. Tumor doubling times were calculated based on these measurements.

A fourth series of animals were administered MitoQ daily at a dose 18 mg/kg from Days -1 to Day +3 (total = 5 doses), combined with daily 2 Gy or sham irradiations on Days 0 to Day +4 (total = 5 doses). Tumor growth was then monitored over time using an electronic caliper to establish growth delay curves and tumor doubling times. At the end of the experiment on Day +98, only animals in the combination treatment group had survived. They were sacrificed using terminal anesthesia and cervical dislocation and tumors were collected, fixed in 4% PFA, and embedded in paraffin. Tumors were processed for immunohistochemistry as described above, except that the primary was a rabbit monoclonal against vimentin (Abcam catalog #AB16700).

**
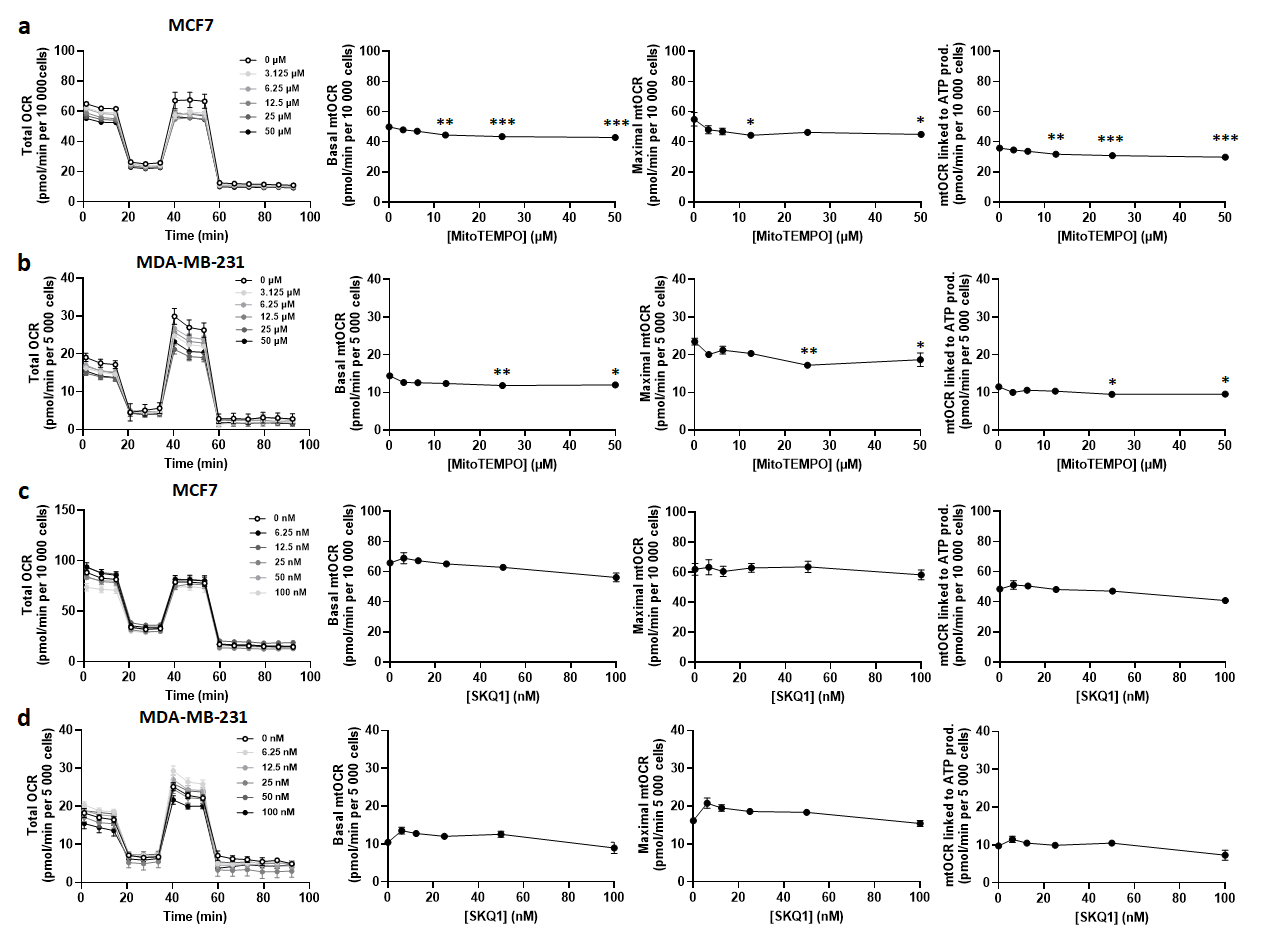
Supplementary Figures**

**Figure S1. MitoTEMPO and SKQ1 do not dose-dependently reduce the oxygen consumption rate of human breast cancer cells.** (**a**, **b**) Human breast cancer cells were treated with increasing doses of MitoTEMPO for 24 h. (**a**) The oxygen consumption rate (OCR) of 10 000 MCF7 cells was measured using Seahorse oximetry. The left graph represents total OCR measurements over time. From Seahorse traces, basal, maximal and ATP-linked mitochondrial OCRs (mtOCRs) were calculated (*n* = 6). (**b**) Same as in (a), but assessing 5 000 MDA-MB-231 cells (*n* = 5-6). (**c**, **d**) Human breast cancer cells were treated with increasing doses of SKQ1 for 24 h. (**c**) The oxygen consumption rate (OCR) of 10 000 MCF7 cells was measured using Seahorse oximetry. The left graph represents total OCR measurements over time. From Seahorse traces, basal, maximal and ATP-linked mitochondrial OCRs (mtOCRs) were calculated (*n* = 6). (**d**) Same as in (c), but assessing 5 000 MDA-MB-231 cells (*n* = 6). All data are shown as means ± SEM. * P < 0.05, ** P < 0.01, *** P < 0.005 by one-way ANOVA with Dunnett’s multiple comparisons test.

**
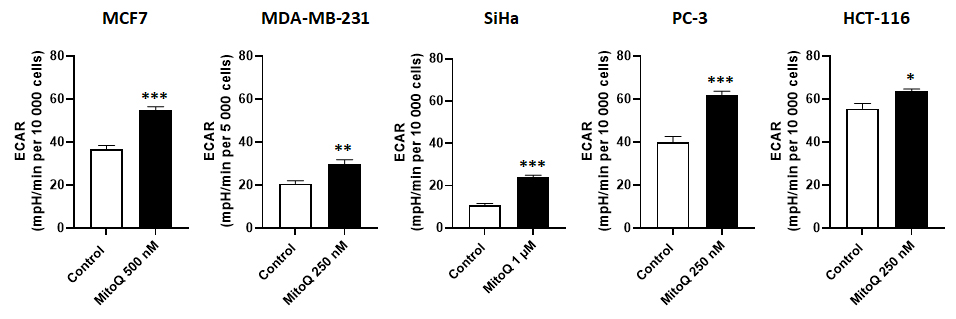
**

**Figure S2. MitoQ increases the extracellular acidification rate of human cancer cells.** The extracellular acidification rate (ECAR) of MCF7 (*n* = 6), MDA-MB-231 (*n* = 6), SiHa (*n* = 4), PC3 (*n* = 4) and HCT116 (*n* = 4) cancer cells was measured using Seahorse pH-metry after a 24 h treatment ± MitoQ at the indicated doses. All data are shown as means ± SEM. * P < 0.05, ** P < 0.01, *** P < 0.001 by Student’s t test.


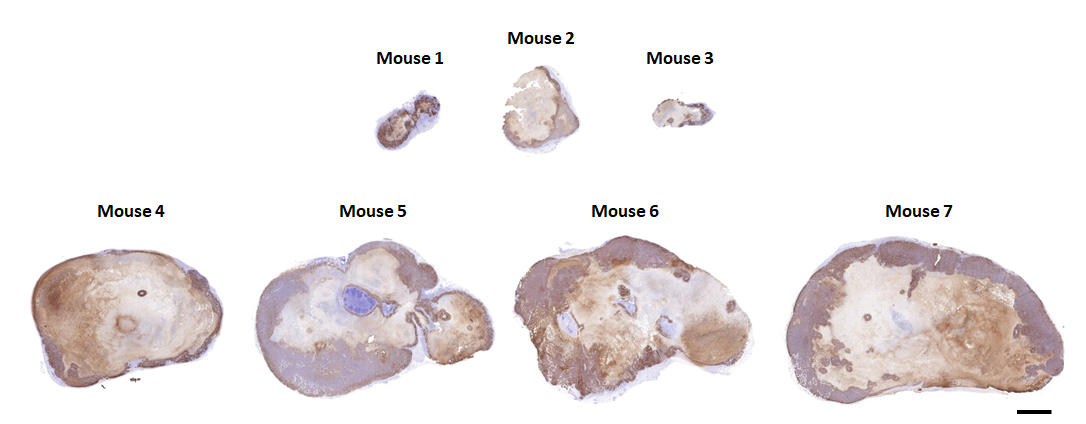


**Figure S3. Some MDA-MB-231 cancer cells survive after a single cycle of MitoQ and irradiation.**  On Day +98 at the end of the experiment displayed in Figure 6f**,** mice in the combination treatment group were sacrificed and the remaining tumors collected. Shown are representative tumor slices for each mouse (*n* = 7) stained for vimentin (brown) and counterstained with hematoxylin (bar = 2 mm).

**Supplementary References**

1 Sonveaux P, Vegran F, Schroeder T, Wergin MC, Verrax J, Rabbani ZN *et al*. Targeting lactate-fueled respiration selectively kills hypoxic tumor cells in mice. *J Clin Invest* **118**, 3930-3942 (2008).

2 d'Hose D, Mignion L, Hamelin L, Sonveaux P, Jordan BF & Gallez B. Statins alleviate tumor hypoxia in prostate cancer models by decreasing oxygen consumption: An opportunity for radiosensitization? *Biomolecules* **12**, 1418 (2022).

3 De Saedeleer CJ, Copetti T, Porporato PE, Verrax J, Feron O & Sonveaux P. Lactate activates HIF-1 in oxidative but not in Warburg-phenotype human tumor cells. *PLoS One* **7**, e46571 (2012).
